# Phytochemistry-Guided Green Synthesis of Antimicrobial Silver Nanoparticles from *Cannabis sativa* Chemovars

**DOI:** 10.3390/ijms27093713

**Published:** 2026-04-22

**Authors:** Fresia M. Silva Sofrás, Sofia Municoy, Jimena Guajardo, Pablo E. Antezana, Nicolás Nagahama, Mariano Cáceres, Pablo L. Santo-Orihuela, Martín F. Desimone

**Affiliations:** 1 Universidad de Buenos Aires, Facultad de Farmacia y Bioquímica, Departamento de Ciencias Químicas, Cátedra de Química Analítica Instrumental, Buenos Aires 1113, Argentina; dra.silvasofras@gmail.com (F.M.S.S.); pablo.e.antezana@gmail.com (P.E.A.); psorihuela@gmail.com (P.L.S.-O.); desimone@ffyb.uba.ar (M.F.D.); 2 Instituto de Química y Metabolismo del Fármaco (IQUIMEFA), CONICET-Universidad de Buenos Aires, Buenos Aires 1113, Argentina; 3 Estación Experimental Agroforestal Esquel, Instituto Nacional de Tecnología Agropecuaria, Esquel 9200, Argentina; 4 Cátedra de Botánica General, Facultad de Ciencias Naturales y Ciencias de la Salud (FCNyCS), Universidad Nacional de la Patagonia San Juan Bosco, Esquel 9200, Argentina; 5 Instituto de Bioquímica y Medicina Molecular (IBIMOL), CONICET-Universidad de Buenos Aires, Buenos Aires 1113, Argentina; 6 CONICET—Centro Científico Tecnológico Patagonia Norte, Esquel 9200, Argentina; 7 Centro de Investigaciones de Plagas e Insecticidas (CIPEIN), UNIDEF, CITEDEF, CONICET, San Juan Bautista de La Salle 4397, Villa Martelli 1603, Argentina; marianocg85@gmail.com

**Keywords:** *Cannabis sativa* L., cannabinoids, chemovar, green synthesis, silver nanoparticles, antimicrobial activity

## Abstract

The phytochemical variability in *Cannabis sativa* L. chemovars represents an underexplored factor in environmentally sustainable nanomaterial production. In this study, three distinct chemovars, (i) High-Δ^9^-Tetrahydrocannabinol (THC) (89% THC), (ii) Balanced (60% Cannabidiol (CBD)), and (iii) High-CBD (89% CBD), were comparatively evaluated to determine their suitability for the green synthesis of silver nanoparticles (AgNPs). Ethanolic inflorescence extracts were used to recover bioactive secondary metabolites; among them, the High-CBD extract exhibited the highest total phenolic (3.34 mg gallic acid equivalent/g) and flavonoid (29.49 mg quercetine equivalent/g) contents, together with superior antioxidant capacity (53.16% 2,2-diphenyl-1-picrylhydrazyl free radical (DPPH) inhibition), indicating enhanced redox potential for nanoparticle formation. The terpene profile of High-CBD showed a dominance of myrcene (21.4%), contributing to the stabilization of the system. Using the High-CBD extract, predominantly spherical nanoparticles of 5 ± 0.9 nm were synthesized and confirmed by UV–vis, EDS, and TEM. The biogenic AgNPs demonstrated significant dose-dependent antibacterial activity, with minimum bactericidal concentration (MBC) of 1.0 mg/mL against *Staphylococcus aureus* and 4.5 mg/mL against *Escherichia coli*. These findings highlight the critical role of chemovar-dependent phytochemical composition and support a phytochemistry-guided approach for developing silver nanoparticles with potential biomedical applications.

## 1. Introduction

Nanomaterials are promising for addressing the major health challenges faced globally due to their particular characteristics, based primarily on their size, distribution, and morphology [1,2]. Metallic nanoparticles (MNPs) are widely used in biomedical fields including drug delivery, imaging, cancer treatment, sensing and gene manipulation [3,4,5]. This widespread use is due to their unique properties, such as a high surface area-to-volume ratio and their ability to be conjugated with antibodies, ligands, and drugs [6,7]. Among all MNPs, silver nanoparticles (AgNPs) stand out in the field of nanotechnology, sparking unlimited interest due to their chemical stability, and most importantly, their antibacterial, antiviral, antifungal, and anti-inflammatory activity [8,9]. They can be part of wound dressings, topical creams, or antiseptic formulations, as their antimicrobial action is explained by generating alterations in the unicellular membranes of bacteria, disrupting their enzymatic activities [10,11]. However, chemically synthesized AgNPs (cAgNPs) used in these products, along with their by-products, can be toxic and harmful to humans and the environment [12]. In an effort to counter these limitations, green synthesis methods have been employed to produce biogenic AgNPs that are more biocompatible with less collateral toxic effects [13,14,15].

Researchers are actively seeking sustainable, eco-friendly alternatives to conventional chemical synthesis. This has significantly advanced green synthesis procedures, particularly plant-mediated synthesis, where plant extracts act as both reducing and stabilizing agents [16,17]. This innovative approach has gained importance as a highly efficient, convenient, fast, ecological, and non-toxic technique for the synthesis of AgNPs [18,19,20]. Various plant components, including barks, roots, stems, fruits, seeds, calluses, peels, leaves, and flowers have been used for nanoparticle synthesis [21,22,23,24,25,26]. The key to this methodology lies in the crucial role of the phytochemicals produced by the plant [27,28]. These natural compounds, mostly water-soluble, include terpenes, polyphenols, flavones, carboxylic acids, carbohydrates, alkaloids, phenolic acids, and proteins, and are responsible for the bioreduction of silver ions during synthesis. Reported studies have emphasized the importance of specific functional groups within these phytochemicals, particularly alkaloids, flavones, and anthracenes, characterized by their –C=C–, –C–O, –C–O–C–, and –C=O– groups, to produce AgNPs [24,29].

In many cases, the reducing agents found in plant extracts serve both protective and stabilizing functions. This eliminates the need to add potentially toxic external chemical compounds [30,31]. AgNPs synthesized from plant extracts are usually more stable and tend to exhibit a wide range of shapes and sizes. Different parameters can be tested during synthesis, including temperature, pH, reaction time, and the concentration of plant extract and silver precursor. This technique also offers an efficient and rapid route, with a higher yield of nanoparticle synthesis, avoiding the complex and slow processes associated with maintaining cell cultures, as often required in other biological methods [13].

Particularly, AgNPs synthesized employing plants exhibit distinct advantages for biomedical applications, owing to the therapeutic properties of secondary metabolites present in the plant extracts used for their synthesis [32,33,34]. For *Cannabis sativa* L., numerous green syntheses of nanoparticles have been reported along with their promising biological activities [35,36,37,38]. The biological activity of AgNPs can be enhanced by the therapeutic properties contributed by *C. sativa*, such as analgesic, antioxidant, and anti-inflammatory properties, but also by the inherent antimicrobial properties of the cannabinoids. It has been reported that cannabinoids, terpenes, and phenolic compounds contribute to antibacterial activity through complementary mechanisms. Cannabinoids, like cannabidiol (CBD), are known to affect bacterial membrane integrity, particularly in Gram-positive strains, leading to increased permeability and cell damage [39]. Terpenes, due to their lipophilic nature, can also disrupt membrane structures and enhance the penetration of other bioactive compounds. Phenolic compounds, including flavonoids and phenolic acids, exhibit antibacterial effects via multiple pathways, such as enzyme inhibition, protein denaturation, and induction of oxidative stress. Overall, the combined presence of these compounds may result in an enhanced therapeutic effect of the green synthesized AgNPs [40,41,42]. However, the aqueous extract used as a reducing agent does not contain cannabinoids or terpenes, which are the most relevant lipophilic molecules of the plant. This is because the parts used for synthesis are not the inflorescences but other plant organs such as roots, bark, seeds, or even leaves [43,44,45].

This study aims to investigate the phytochemical potential of three distinct chemovars of *C. sativa* for the efficient green synthesis of AgNPs using ethanolic extracts from their inflorescences. In contrast to previous research that predominantly employed leaf material or other organs [43,44,45], this work focuses on inflorescences due to their richer content of cannabinoids and other bioactive secondary metabolites. This approach pursues to emphasize the relevance of full-spectrum compositions in cannabis extracts, particularly considering the reported ‘entourage effect’ [46], where synergistic interactions among metabolites may enhance biological activity.

In this context, the selection of *C. sativa* inflorescences is based on their unique phytochemical diversity. Specifically, cannabinoids like CBD possess phenolic hydroxyl groups that efficiently reduce silver ions (Ag^+^) to metallic silver (Ag^0^), while the complex chemical structure of these metabolites and associated terpenes provides natural capping and steric stabilization. As a recognized medicinal plant, utilizing these inflorescence-derived compounds imparts an intrinsic therapeutic ‘added value’ to the nanoparticles. By leveraging the synergistic potential between the plant’s bioactive constituents (e.g., anti-inflammatory or analgesic properties) and the antimicrobial silver core, this method offers an enhanced potential for biomedical applications compared to traditional chemical synthesis. Ultimately, the objective of this comprehensive profiling is to identify which chemovar presents the most promising phytochemical profile for the sustainable and efficient production of bioactive nanomaterials.

## 2. Results and Discussion

### 2.1. Cannabinoids Quantification

**Figure 1:**
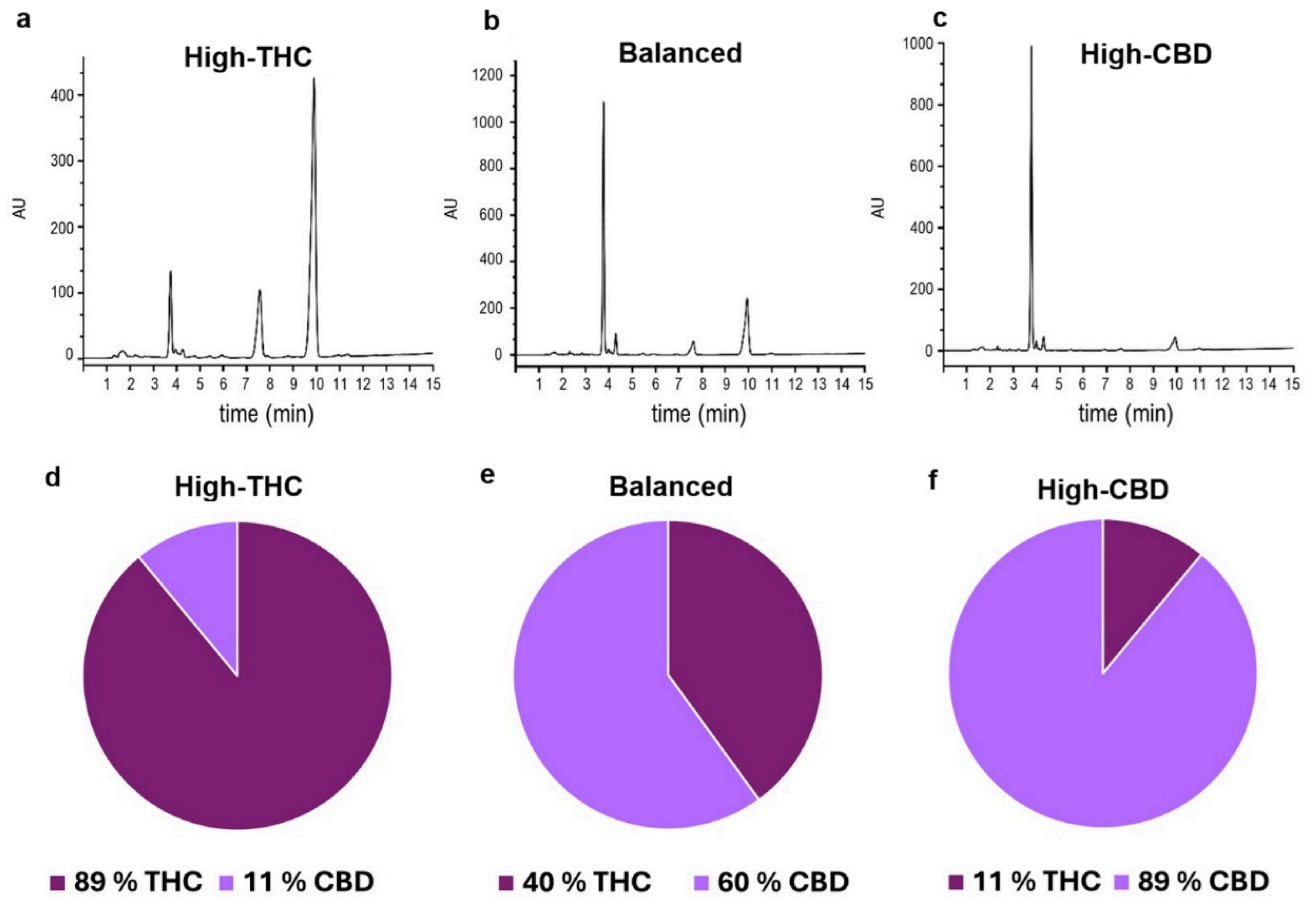
Chromatograms and corresponding pie charts illustrating the CBD and THC content in the three cannabis varieties/chemovars: High-THC (**a**,**d**), Balanced (**b**,**e**), and High-CBD (**c**,**f**).

High Performance Liquid Chromatography (HPLC) was used to quantify cannabinoids content of the three cannabis varieties. Due to their high cannabinoid concentration and viscosity, the semi-liquid resins were pre-diluted in methanol (MeOH) to prevent column damage and ensure analytical precision. Figure 1 presents the corresponding chromatograms for each variety showing differences among the three types in terms of cannabinoid dominance. The High- THC variety was characterized by the presence of 89% of THC and 11% of CBD, Balanced variety had 40% of THC and 60% of CBD and High-CBD variety showed 89% of CBD and 11% of THC. Although the relative cannabinoid concentrations (Table 1) did not strictly meet the percentage standards for chemotype classification (all varieties could be classified as chemotype II) significant differences in cannabinoid dominance were observed among the three varieties (*p*-value < 0.05). Tukey’s post hoc test identified each variety as a distinct group, confirming that all three resins are significantly different from one another.

### 2.2. Terpene Profile

**Figure 2:**
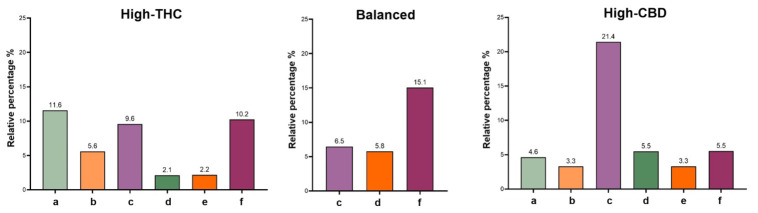
Relative percentage (%) of most abundant (>2%) terpenes for the three chemovars. (**a**) α-pinene, (**b**) β-pinene, (**c**) myrcene, (**d**) limonene, (**e**) linalool, and (**f**) β-caryophyllene.

Gas Chromatography (GC) analysis of the terpene profile of High-THC, Balanced and High-CBD detected the presence of the monoterpenes myrcene and linalool in all varieties, as well as the sesquiterpene β-caryophyllene (Figure 2). The results, expressed as the relative abundance of each terpene, indicate some variability in abundance among the varieties. For this study, only compounds with a relative abundance of at least 2% were considered. In the High-CBD variety, the predominant component is myrcene, while in Balanced, it is β-caryophyllene. The High-THC variety, on the other hand, is characterized primarily by α-pinene.

### 2.3. Antioxidant Activity

**Figure 3:**
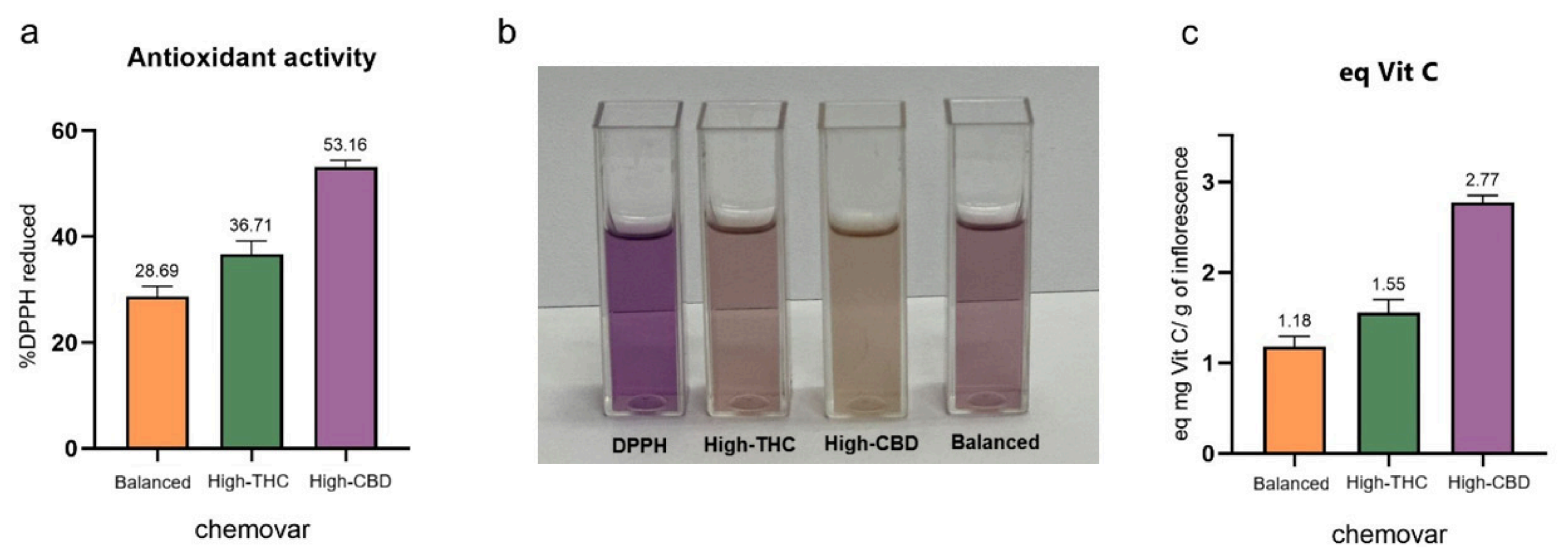
Antioxidant activity for each analyzed variety (High-CBD, Balanced, and High-THC): (**a**) % DPPH neutralized per 100 μL of extract, (**b**) Visual data of DPPH reacting to the chemovars, (**c**) mg Vitamin C equivalent per gram of inflorescence. Values are presented as means ± SD (*n* = 3).

In ascending order of antioxidant activity, the varieties ranked as follows: Balanced, High-THC, and High-CBD, meaning the variety with the highest CBD concentration (High-CBD) exhibited the greatest reducing capacity (Figure 3a). In addition to recording differences in DPPH radical scavenging capacity, there were significant differences in the antioxidant activity of High-CBD, Balanced, and High-THC (Figure 3a). According to Tukey’s post hoc comparison, two separate groups were observed, highlighting significant differences between the High-CBD variety and a group comprising Balanced and High-THC (which were not significantly different from each other in terms of antioxidant activity). Visually, differences were also noticeable in the color shift in neutralized DPPH for each variety (Figure 3b), with a more intense yellow coloration observed in the High-CBD variety. Furthermore, as previously mentioned, the antioxidant activity was also calculated as the equivalent amount of ascorbic acid (Vitamin C), in mg per gram of plant material (Figure 3c).

### 2.4. Total Phenol and Flavonoid Content

**Figure 4:**
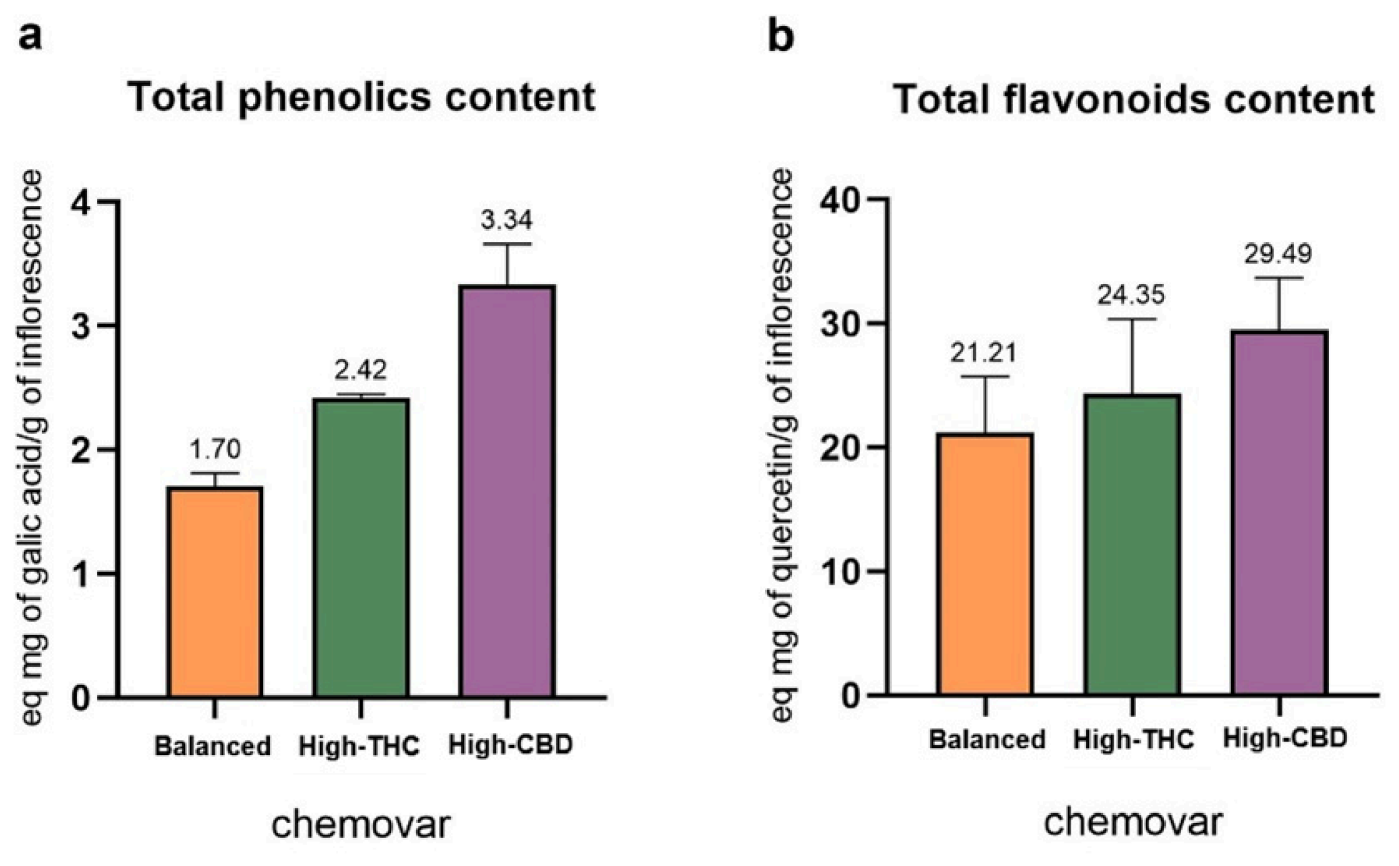
(**a**) Total phenol content expressed as μg gallic acid equivalent and (**b**) total flavonoid content expressed as mg quercetin equivalent, per gram of inflorescence for the analyzed chemovars. Values are presented as means ± SD (*n* = 3).

Regarding the total phenol content, statistically significant differences were observed between varieties (*p*-value < 0.0001). The inflorescences of the High-CBD plants showed a higher total phenol content compared to the Balanced and High-THC varieties (Figure 4a). The equivalents of mg of gallic acid per gram of plant ranged from 1.70 to 6.91. On the other hand, when analyzing the total flavonoid content, no significant differences were observed between the different varieties. This indicates that the varieties are similar in terms of polyphenol abundance. The equivalents of mg of quercetin per gram of inflorescence ranged from 21.21 to 35.44, according to quercetin calibration curve (Figure 4b).

### 2.5. Multivariate Analysis for Chemovar Selection, Biplot Interpretation

**Figure 5:**
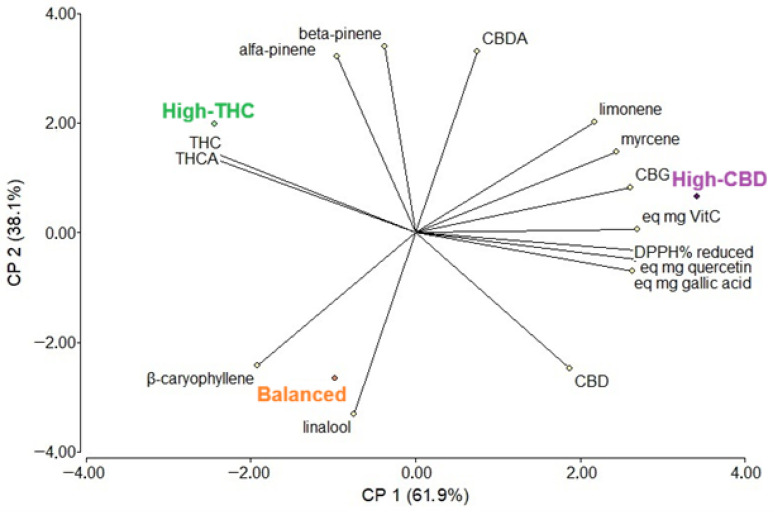
Biplot based on Principal components multivariate analysis (PCMA) of all measured parameters for the three chemovars, with data normalized and standardized prior to analysis.

Principal component multivariate analysis (PCMA), along with the Biplot graph, has been recently proposed for the comprehensive study of cannabis varieties [45]. The resulting biplot displays the distribution of the samples in a reduced dimensional space, where the first two principal components account for 100% of the total variance. A Biplot in a PCMA is a valuable tool for exploratory analysis of multiple measured parameters (Figure 5). Each colored point represents a chemovar (High-CBD, Balanced, High-THC), and the vectors indicate the direction and strength of each variable’s contribution. Variables pointing in similar directions are positively correlated, while those pointing in opposite directions are negatively correlated. This approach enables simultaneous visualization of inter-variable relationships, and it is useful to identify patterns, trends, and data clusters for a more objective selection of plant material based on multiple phytochemical criteria.

Based on this analysis, the High-CBD variety emerges as the most suitable candidate for the green synthesis of AgNPs. This chemovar clustered strongly with high levels of non-psychoactive cannabinoids (CBD, CBDA, CBG), together with enhanced antioxidant activity (expressed as DPPH% reduction and vitamin C equivalents), total phenolic content (gallic acid equivalents), and total flavonoid content (quercetin equivalents). These compounds play a key role as natural reducing and capping agents during the synthesis of AgNPs [17,47,48]. Phytochemicals present in *C. sativa* exhibit strong antioxidant activity, promoting the reduction of metal ions while undergoing structural transformations, such as the conversion of enol groups (alkene + alcohol) into more stable keto forms (aldehydes or ketones) via proton transfer [39]. Additionally, these molecules inhibit nanoparticle agglomeration by adsorbing onto the AgNPs surface and forming a protective layer that counteracts intrinsic attractive forces. This behavior explains the superior efficiency of the High-CBD chemovar in the sustainable synthesis and stabilization of silver nanoparticles.

### 2.6. Synthesis of Silver Nanoparticles (AgNPs)

**Scheme 1:**
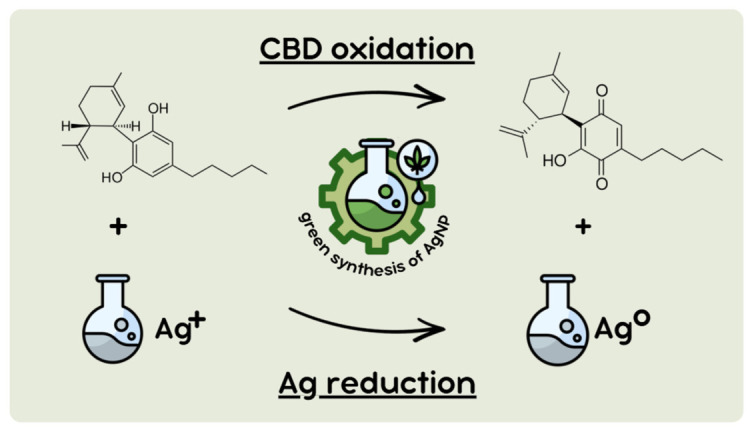
Proposed mechanism for the formation of AgNPs: the oxidation of CBD promotes the reduction of Ag^+1^ to Ag^0^, leading to nanoparticle formation.

The High-CBD variety was characterized by a predominant presence of the cannabinoid CBD, a compound widely recognized for its therapeutic properties and its antimicrobial activity, particularly relevant to this study. Moreover, this variety exhibited significantly higher antioxidant activity and greater total phenolic and flavonoid content compared to the other varieties evaluated. These attributes are of particular interest for the synthesis of MNPs, due to their potential medicinal properties, which may act synergistically with the known antimicrobial effects of AgNPs [9,49,50]. At the same time, these biomolecules can serve as reducing and stabilizing agents during synthesis [13]. By oxidation of the biocompounds, the reduction of silver ions leads to the formation of AgNPs. Given the high concentration of CBD, one of the possible mechanisms involves its oxidation to cannabidiolquinone (CBDQ), which promotes the reduction of Ag^+^ to Ag^0^ (Scheme 1).

One of the challenges in synthesizing the silver nanoparticles was optimizing the final experimental conditions. The primary difficulty occurred when using water as the reaction medium. Under these conditions, it was not possible to characterize or visualize the formation of NPs. This may be due to the low solubility of the cannabinoids present in the ethanolic extract in water, which can be attributed to their high lipophilicity. These molecules play a significant role in reducing Ag^+^ ions during nanoparticle synthesis, and their absence in the medium hinders the efficiency of the process. This issue was resolved by using absolute ethanol as the reaction medium, which is also known as an environmentally friendly solvent.

### 2.7. Characterization of AgNPs

**Figure 6:**
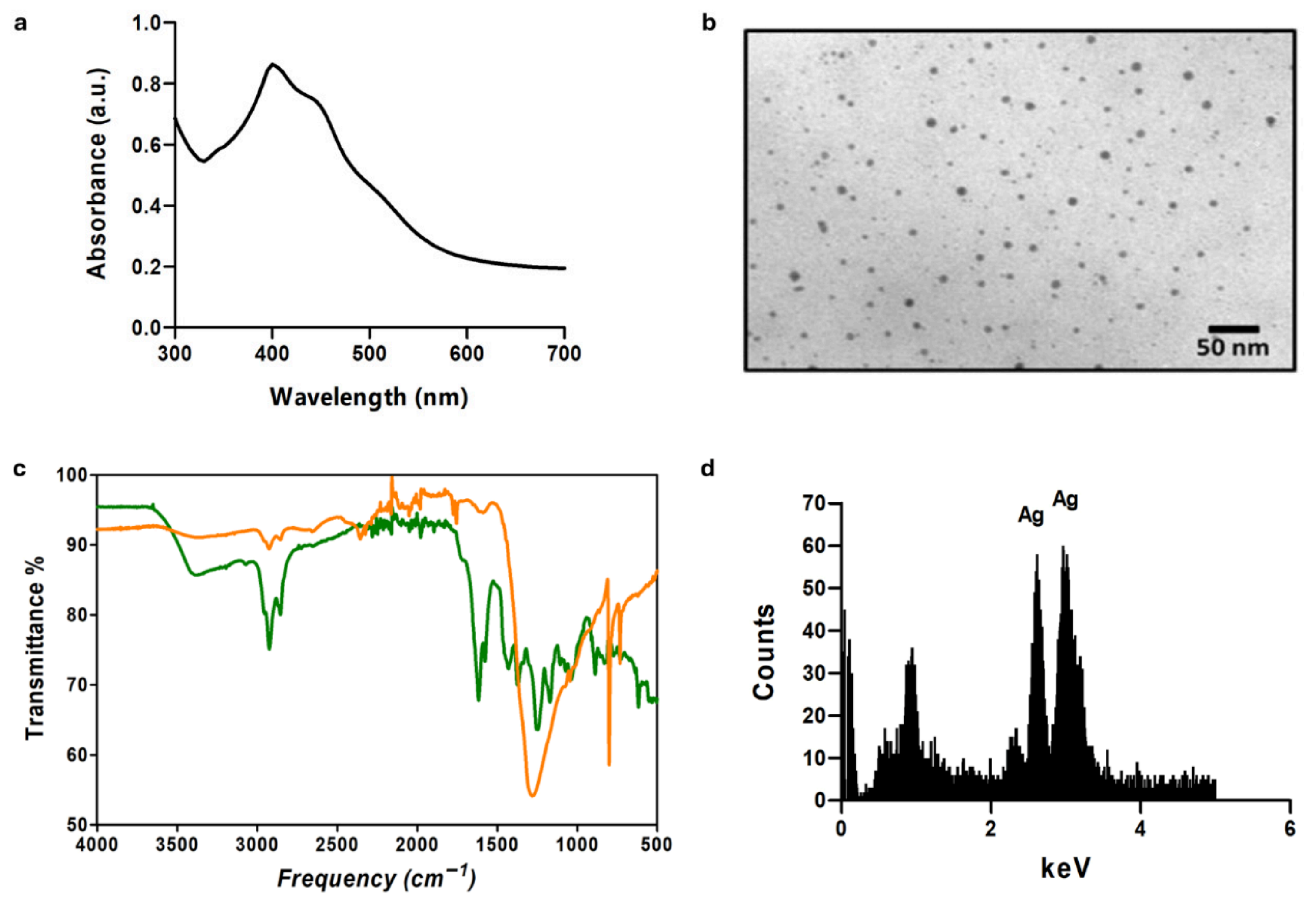
Characterization of green synthesized AgNPs. (**a**) UV-vis spectrum of AgNPs shows the surface plasmon resonance characteristic of silver nanoparticles. (**b**) TEM images of AgNPs, using a magnification of 140,000×. (**c**) FTIR analysis of ethanolic extract of High-CBD (green) and green synthesized AgNPs (orange). (**d**) EDS analysis of AgNPs showing characteristic peaks of Ag.

One of the confirmatory assays for the formation of AgNPs involved the analysis of the UV-visible spectrum (Figure 6a), as these NPs are known to exhibit a strong absorption between 400 and 450 nm, corresponding to the characteristic surface plasmon resonance [9,51]. The peak maximum at around 400 nm also indicates that the particles are of nanometric size in agreement with Transmission Electron Microscopy (TEM) images (Figure 6b). Figure 6b exhibits well-dispersed spherical nanoparticles of 5.0 ± 0.9 nm. This proves that phytocompounds form an effective coating on the inorganic silver surface, thereby preventing nanoparticle self-aggregation. This was confirmed by Fourier Transform Infrared (FTIR) analysis (Figure 6c). In fact, the FTIR spectrum of AgNPs exhibits two distinct peaks at 2922 and 2850 cm^−1^, corresponding to the asymmetric C–H stretching vibrations of alkanes present in the High-CBD extract [52]. Additionally, the signal at 1598 cm^−1^, attributed to the C=C stretching vibration of aromatic rings, along with the broad band at 1283 cm^−1^ related to C–OH bending, further confirms the formation of a green coating. Furthermore, the peaks at 795 and 731 cm^−1^ show the presence of =C–H bending aromatic groups. These functional groups, derived from the plant extract, play a key role in the reduction and capping of the silver nanoparticles [45]. Notably, the attenuation of the band in the 3500 cm^−1^ region (phenolic O–H stretching) indicates the oxidation of phenolic hydroxyl groups. Complementary Energy-Dispersive X-ray Spectroscopy (EDS) analysis further confirmed the composition of the nanoparticles, providing an elemental analysis of the sample. The EDS spectrum of Figure 6d showed a strong signal at 3 keV, indicating the presence of silver [53,54].

### 2.8. Antimicrobial Activity

**Figure 7:**
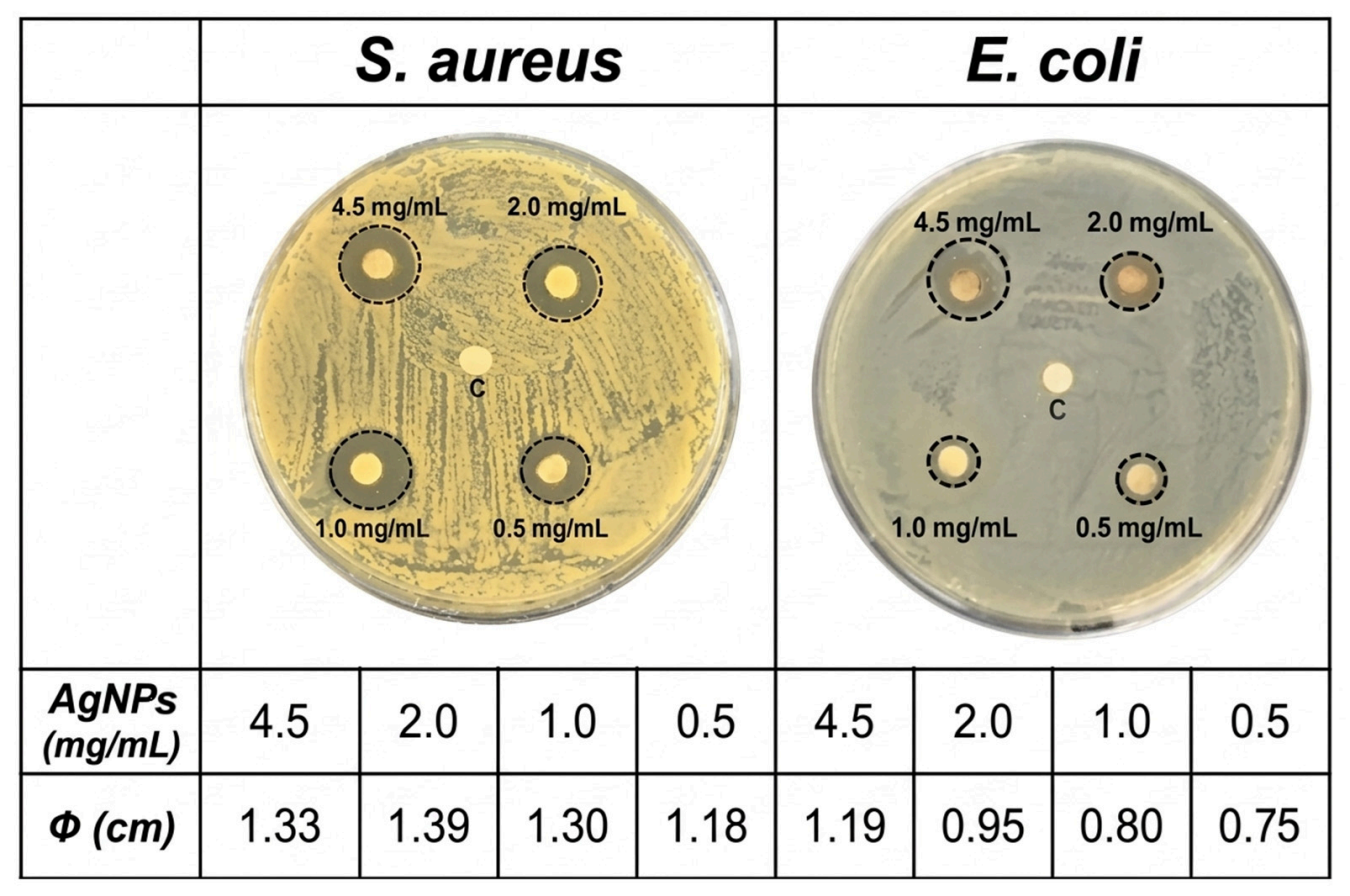
Antibiograms of AgNPs against Gram-positive (*S. aureus*) and Gram-negative (*E. coli*) bacteria. C corresponds to control without AgNPs. Փ refers to the diameter of the bacteria-free inhibition zones expressed in cm.

Antimicrobial activity of AgNPs was evaluated against two bacterial strains, *S. aureus* and *E. coli*, using both disk diffusion and dilution methods. For the antibiogram assays, a bacterial suspension was uniformly spread onto agar plates, and sterile filter paper discs impregnated with 50 μL of different AgNPs concentrations (4.5, 2.0, 1.0, and 0.5 mg/mL) were placed on the agar surface. After 24 h of incubation at 37 °C, the diameters of the inhibition zones surrounding each disc were measured. As shown in Figure 7, all tested concentrations exhibited antibacterial activity against both strains, evidenced by the presence of clear and well-defined inhibition halos around the discs. The size of these zones increased with nanoparticle concentration, indicating a dose-dependent antimicrobial effect.

A comparative analysis revealed that *S. aureus* was more susceptible to AgNPs treatment than *E. coli*, as demonstrated by consistently larger inhibition zones at equivalent concentrations. This difference in sensitivity may be attributed to structural variations in the bacterial cell wall, particularly the outer membrane present in Gram-negative bacteria such as *E. coli*, which can act as an additional barrier to nanoparticle penetration [9].

Furthermore, the bactericidal activity of AgNPs was confirmed through liquid medium assays. The minimum bactericidal concentration (MBC) for *S. aureus* was determined to be 1.0 mg/mL, at which no viable bacterial growth was detected after plating. In contrast, *E. coli* required a higher concentration to achieve a bactericidal effect, with the MBC observed at 4.5 mg/mL. These findings reinforce the greater resistance of *E. coli* and highlight the concentration-dependent efficacy of AgNPs as an antimicrobial agent.

## 3. Materials and Methods

### 3.1. Sample Origin

The samples utilized were cultivated and provided by the non-governmental organization (NGO) “Mamá Cultiva”. The NGO provided 15 g of inflorescences from three different stabilized variety of *Cannabis sativa* L. These genotypes were originally established from specific seed lineages and maintained as mother plants to ensure a standardized and reproducible cultivation process. For scientific clarity, the varieties are identified according to their predominant cannabinoid profiles as determined by preliminary HPLC screening: High-THC (a variety characterized by a high relative abundance of Δ^9^-Tetrahydrocannabinol), Balanced (a variety characterized by an intermediate ratio of major cannabinoids), and High-CBD (a variety characterized by a high relative abundance of cannabidiol).

### 3.2. Cannabinoids Quantification

To quantify cannabinoids, a resin was extracted from the cannabis flowers provided by Mamá Cultiva [55]. For this, 15 g of dried cannabis inflorescences were extracted with 1 L of triple-distilled grain ethanol under continuous stirring for 10 min. After two filtration steps, the ethanol was evaporated, and the resulting resin was collected in syringes for later dilution. Then, 1–2 mg of resin was weighed and diluted with 10 mL of methanol. The suggested approximate concentration for HPLC analysis was 0.1–0.2 mg/mL of resin.

Cannabinoids were quantified by the validated HPLC-UV method according to Silva et al. [56]. The chromatographic conditions are summarized in Table 2. The HPLC-MWD liquid chromatography system (Agilent Technologies, Santa Clara, CA, USA) consisted of a 1260 VL binary pump coupled to a 1260 MW UV detector with eight wavelengths. Samples were injected using an Agilent injector (20 μL loop). Chromatographic system control was carried out using OpenLab CDS ChemStation software (version. C.01.08).

A solution containing 33.3 μL/mL of the seven main cannabinoids—CBDA (cannabidiolic acid), CBG (cannabigerol), CBD, CBN (cannabinol), Δ^9^-THC, CBC (cannabichromene), and THCA (tetrahydrocannabinolic acid)—was used as a reference standard. The CBDA (lot No. A0168639), CBG (lot No. A0158938), Δ^9^-THC (lot No. A0159145), CBC (lot No. A0162635), and THCA (lot No. 0604183) standards (1 mg/mL in methanol) were obtained from Restek^®^ Laboratories (Bellefonte, PA, USA). The CBD standard was provided by Enecta Laboratories (Amstelveen, The Netherlands) while the CBN was isolated through preparative chromatography, resulting in a chromatographic purity of 98.0%.

### 3.3. Characterization of Terpenes Profile

Terpenes profile of each chemovar was analyzed by gas chromatography-mass spectrometry (GC-MS). For this, hexane extracts were prepared for injection the assay. A total of 50 mg of inflorescence was ground and extracted with 1 mL of hexane HPLC grade from MilliporeSigma™. The mixture was then sonicated for 15 min and filtered through a 0.22 μm membrane filter. Terpenes identification followed Ibrahim et al. procedures [57] and was performed using gas chromatography coupled with mass spectrometry (GC-MS). The equipment used was a Shimadzu GC-2010 gas chromatograph with a QP2010 quadrupole mass spectrometer. A non-polar DB-5MS column (J&W Scientific, Agilent Technologies) was used, with the following dimensions: 30 m length × 0.25 mm internal diameter and 0.25 μm film thickness. Helium was used as the carrier gas at a constant flow rate of 1 mL/min. The injector temperature was set at 250 °C, with a split ratio of 15:1. Two 2 μL injections were performed for each sample. The oven temperature program was as follows: initially held at 50 °C for 2 min, followed by an increase of 2 °C/min to 85 °C, and then a further increase of 3 °C/min up to 165 °C. The mass spectrometer was operated with an ion source temperature of 230 °C, an interface temperature of 280 °C, and a detector temperature of 150 °C. Compound identification was carried out using the Wiley8 and NIST08 libraries.

### 3.4. Sample Preparation for Antioxidant Activity, Total Phenolic Content and Total Flavonoid Content Measurements

An ethanolic extract was prepared by macerating 500 mg of ground inflorescence in 10 mL of 96% ethanol for 7 days. After this period, the mixture was filtered and adjusted to a final volume of 10 mL.

### 3.5. Antioxidant Activity

The antioxidant activity was measured using the DPPH• (2,2-diphenyl-1-picrylhydrazyl free radical) colorimetric method based on a color change from violet to yellow upon the reaction of DPPH• with an antioxidant compound [9,58]. For this, 20 μL of each extract was incubated with 3 mL of the ethanolic solution of DPPH• (30 mg/mL). The absorbance at 517 nm was measured before and after the reaction using a spectrophotometer Spectrum SP 2000 UV (Shanghai Spectrum Instruments Co., Ltd., Shanghai, China). The antioxidant capacity was calculated as a percentage of inhibition using the following equation:

$$\%\;Inhibition=\left[1-\left(\frac{Abs\;sample}{Abs\;DPPH\;solution}\right)\right]\times 100$$

The assay was performed in triplicate and the results were then expressed as % DPPH reduced per 100 mL ± SD and as mg vitamin C equivalents per gram of inflorescence using a calibration curve of vitamin C.

### 3.6. Total Phenolic Content

The total phenolic content was determined using the Folin–Ciocalteu reagent, which changes from yellow to blue upon reduction by phenolic compounds. An aqueous solution of 16% Na_2_CO_3_ was prepared by dissolving 8 g of Na_2_CO_3_ in 50 mL of water. The pure Folin–Ciocalteu reagent was diluted as follows: 5 mL of reagent was diluted to 20 mL with distilled water.

In each spectrophotometric cuvette, 2 mL of distilled water was pipetted, followed by 80 μL of each extract, and finally 0.8 mL of the Na_2_CO_3_ solution, allowing the reaction to proceed for 20 min. Absorbance was measured at 765 nm (Spectrum SP 2000 UV), and the results were expressed as mg gallic acid equivalents per gram of inflorescence using a calibration curve of gallic acid.

### 3.7. Total Flavonoid Content

Total flavonoids were measured by the chelation of flavonoids with aluminum chloride, which was quantified spectrophotometrically at 425 nm (Spectrum SP 2000 UV). A 5% ethanolic solution of AlCl_3_ was prepared by dissolving 3 g of anhydrous AlCl_3_ in 60 mL of 96% ethanol.

In each cuvette, 1.5 mL of ethanol was first added, followed by 40 μL of each sample corresponding to variety and extract type, and finally 1 mL of the AlCl_3_ solution. The mixture was incubated for 20 min at room temperature. Results were expressed as mean mg quercetin equivalents per gram of inflorescence ±SD from triplicate experiments.

### 3.8. Data Analysis for Suitable Chemovar Selection

Statistical analyses were performed using Infostat v. 2015 [59]. To determine significant differences between the resins in terms of cannabinoid content, one-way ANOVA (α = 0.05) was performed to evaluate the significance of the differences between resins. Tukey’s test (*p* < 0.05) was applied for *a posteriori* comparison of each pair of means. Additionally, to compare antioxidant activity, total phenol content, and flavonoid content, a nested-design ANOVA was performed to compare the results for each parameter, considering both extract type and variety. All analyses were conducted using a significance level of *p*-value < 0.05, where the null hypothesis (*p*-value > 0.05) indicates no significant differences (*n* = 3 for all essays).

Furthermore, Principal Component Analysis (PCA) was applied to explore patterns and reduce dimensionality among the measured variables across different cannabis varieties. This approach allowed us to identify the most strongly associated variety with favorable parameter profiles, guiding the selection of the optimal candidate for nanoparticle synthesis. A biplot graph based on the PCA obtained was constructed to summarize the observations for the three varieties regarding the standardization of measured parameters.

PCA and the corresponding Biplot visualization were performed using InfoStat software (version 2015, Universidad Nacional de Córdoba, Córdoba, Argentina).

### 3.9. Synthesis of Silver Nanoparticles (AgNPs)

For the synthesis of AgNPs, 1 mL of a 0.1 M AgNO_3_ solution was mixed with 100 μL of cannabis extract, respectively, in an ethanolic medium, with a final volume of 10 mL. For this, 0.1 mL of cannabis extract was mixed with 8.9 mL of ethanol and the 0.1 M AgNO_3_ solution was added dropwise. The reaction medium was kept at 50 °C under agitation for 10 min. Once the reaction was complete, the resulting suspension was stored at room temperature in the dark. To determine the concentration of the AgNPs, the entire suspension was dried in an oven at 60 °C, and the resulting powder was weighed. By relating the mass of the powder to the initial volume, the concentration of the AgNPs was calculated.

#### 3.9.1. Characterization of AgNPs

The formation of AgNPs was confirmed by analyzing the surface plasmon resonance by UV-visible spectrophotometry using a Jasco V-730 spectrophotometer (JASCO, Tokyo, Japan), controlled by SpectraManager software (version 2.9). The morphology and size of the AgNPs were evaluated by transmission electron microscopy (TEM) using a Zeiss EM109T electron microscope (Carl Zeiss, Oberkochen, Germany). For this, a drop of the AgNPs suspension was placed on carbon-coated copper grids and allowed to dry for a few minutes. The average size of the AgNPs was calculated using ImageJ software (version 1.52f) based on TEM images. Additionally, the presence of silver was confirmed by Energy-Dispersive X-ray Spectroscopy (EDS). Fourier transform infrared (FTIR) spectra of green synthesized AgNPs, chemically synthesized AgNPs and ethanolic extract of High-CBD were obtained over the range of 4000–500 cm^−1^, using an FTIR-Raman Nicolet iS 50 (Thermo Fisher Scientific, Waltham, MA, USA). For this, an aliquot of the samples was dried under a nitrogen flow, and the powder was then placed on the attenuated total reflection accessory of the spectrometer and pressed to record the spectra.

#### 3.9.2. Antimicrobial Activity of AgNPs

The bactericidal activity of AgNPs was evaluated against Gram-negative bacteria (*Escherichia coli*) and Gram-positive bacteria (*Staphylococcus aureus*). Bacteria were cultured overnight at 37 °C in Luria Bertani (LB) medium (yeast extract, 5 g/L; NaCl, 10 g/L; tryptone, 10 g/L), and the cultures were diluted to obtain a concentration of 1 × 10^6^ Colony Forming Units (CFU)/mL. The AgNP powder was resuspended in 2 mL of distilled water, yielding a suspension with a concentration of 4.5 mg/mL of AgNPs.

The bactericidal activity was studied using two different methods: the disc diffusion method for antibiograms and the dilution method [9]. In the first case, 100 μL of each bacterial suspension (from a 1:1000 dilution) was spread on a Petri dish containing nutrient agar using a Drigalski spatula and incubated 10 min at room temperature. The AgNP suspension was then serially diluted to obtain concentrations of 0.5, 1.0, and 2.0 mg/mL. Paper discs were impregnated with 50 μL of each solution and placed on the plate. The inhibitory effect of the different concentrations of AgNPs were evaluated after 24 h by measuring the diameter of the clear zones around the discs.

Additionally, the bactericidal activity of the nanoparticles was evaluated in liquid medium. For this purpose, 100 μL of different concentrations of AgNPs were incubated with 100 μL of a 1:1000 bacterial suspension of *S. aureus* and *E. coli* in 800 μL of LB medium at 37 °C for 24 h. The control consisted of 900 μL of medium and 100 μL of each bacterial suspension. After incubation, 20 μL of each sample was plated onto agar Petri dishes, and bacterial growth was assessed after an additional 24 h of incubation at 37 °C.

## 4. Conclusions

This study delivers a comprehensive and integrative phytochemical assessment of three *Cannabis sativa* L. chemovars (High-THC, Balanced, and High-CBD), demonstrating that chemovar-specific metabolite composition is a decisive factor in the development of functional nanomaterials. By combining cannabinoid and terpene profiling with antioxidant, phenolic, and flavonoid analyses, a robust and exhaustive comparison was achieved, enabling a rational and evidence-based selection of plant material.

Among the evaluated chemovars, the High-CBD variety emerged as the most suitable candidate, exhibiting superior redox potential and a phytochemical profile enriched in bioactive compounds capable of acting as both reducing and stabilizing agents. This selection was further supported by multivariate analysis, highlighting the value of a holistic, data-driven approach in nanomaterial design.

The successful synthesis of small, stable, and biologically active silver nanoparticles using the High-CBD extract underscores the potential of phytochemistry-guided strategies in advancing green nanotechnology for biomedical applications. Notably, the intrinsic bioactivity of the plant-derived compounds, combined with the well-established antimicrobial properties of AgNPs, positions these nanomaterials as promising candidates for future biomedical developments, particularly in antimicrobial therapies and infection control. Overall, this work contributes to recent advances in sustainable nanotechnology by bridging phytochemical characterization with the rational design of bioactive nanomaterials.

## Figures and Tables

**Table 1:** Cannabinoids concentration (mg/mg of resin) in High-THC, Balanced and High-CBD varieties, expressed as mean ± SD.

Cannabinoids (mg/mg Resin)	High-THC	Balanced	High-CBD
CBD	4.9 × 10^−3^ ± 1.0 × 10^−5^	0.02 ± 1.002 × 10^−4^	0.02 ± 1.0 × 10^−5^
THC	0.12 ± 1.761 × 10^−4^	0.05 ± 2.172 × 10^−4^	0.01 ± 9.272 × 10^−5^
THCA	0.42 ± 1.062 × 10^−4^	0.20 ± 4.7172 × 10^−3^	0.06 ± 6.711 × 10^−4^
CBDA	0.07 ± 8.399 × 10^−4^	0.36 ± 6.733 × 10^−4^	0.56 ± 1.591 × 10^−3^
CBG	0.01 ± 1.275 × 10^−4^	0.01 ± 1.179 × 10^−4^	0.02 ± 1.006 × 10^−4^

**Table 2:** Summary of validated chromatographic conditions for the quantification of cannabinoids by HPLC-UV.

Column	Guard ULTRA Security C18 (2.1 mm × 4.6 mm × 5 μm). Phenomenex^®^ Kinetex XB-C18 (250 mm × 4.6 mm × 5 μm)						
Mobile phase	A: 0.085% (*v*/*v*) phosphoric acid in water (Milli-Q water) B: 0.085% (*v*/*v*) phosphoric acid in acetonitrile						
Gradient	min	0	6	14	19	21	25
	% A	25	25	15	5	25	25
	% B	75	75	85	95	75	75
Flow rate	1.6 mL/min						
Temperature (°C)	35 °C						
Detection	UV 220 nm						

## Data Availability

The original contributions presented in this study are included in the article. Further inquiries can be directed to the corresponding author.
